# A Review on the Mechanism of Tuina Promoting the Recovery of Peripheral Nerve Injury

**DOI:** 10.1155/2021/6652099

**Published:** 2021-07-01

**Authors:** Zhifeng Liu, Hourong Wang, Tianyuan Yu, Yi Jiao, Yingqi Zhang, Di Liu, Yajing Xu, Qian Guan, Mengqian Lu

**Affiliations:** ^1^School of Acupuncture-Moxibustion and Tuina, Beijing University of Chinese Medicine, Beijing 102488, China; ^2^Acupuncture Department, Oriental Hospital of Beijing University of Chinese Medicine, Beijing 100078, China

## Abstract

Tuina, as one of the characteristic external therapies of Traditional Chinese Medicine (TCM), has been used to treat the disease caused by peripheral nerve injury (PNI) for thousands of years. An increasing number of clinical trials and animal experiments have demonstrated that tuina can improve the symptoms and promote the recovery of damaged nerves. This review focuses on the mechanistic studies of tuina in promoting the recovery of PNI, which might provide a neurobiological foundation for the effects of tuina. Although many mechanisms underlying the effects of tuina on nerve repair have been identified, there are still many unknown problems, such as the key substance or way for tuina to work, so further investigation is warranted.

## 1. Introduction

Peripheral nerve injury (PNI) is damage to the peripheral nerve plexus, nerve trunk, or its branches due to various reasons such as stretching, cutting, compression, or ischemia, which can lead to motor, sensory, and autonomic dysfunction [1]. Unlike the central nervous system (CNS), the peripheral nervous system (PNS) has a certain capability to regenerate and repair after injury. However, since the repair speed is slow [2, 3], timely interventions should be given to accelerate nerve repair after PNI. Commonly used repair methods include surgery, nerve grafting, rehabilitation, medications, and physiotherapy [4], of which surgery is the main therapy for PNI. Although great progress has been made in surgery and nerve regeneration in recent years, functional recovery is still unsatisfactory [5, 6].

Tuina, as a complementary and alternative medical treatment, is used worldwide due to its advantages in terms of efficacy and safety. Tuina has been proved to be effective in many clinical trials. For example, Ma et al. [7] used tuina to treat 40 patients with obstetrical brachial plexus injury. After 90-day treatment, the basic movement, coordination, and Obstetrical Brachial Plexus Injury Function (OBPIF) scores of the patients were significantly improved (*P* < 0.05), the markedly effective rate reached 70%, and the effective rate was 100%, which demonstrates that tuina has a good effect on obstetrical brachial plexus injury. A randomized controlled trial showed that tuina was effective, safe, and relatively cost-effective in the treatment of chronic neck pain [8]. A meta-analysis involving 121 studies with 13,075 patients showed that, compared with traction and Chinese herbs, tuina and acupuncture were more effective in treating lumbar disc herniation (LDH) [9]. Another meta-analysis showed that massage was more effective than placebo and some other therapies in the treatment of nonspecific low back pain [10].

Compared with other repair methods, such as surgery and medications, tuina has the advantage of outstanding curative effects, economy, security, and fewer side effects. It has been increasingly used to treat PNI-related diseases, and its repair mechanism has also made certain progress in recent years. Thus, this review summarizes the results of basic experiments to explain the mechanism by which tuina promotes the repair of PNI.

## 2. Tuina Can Improve Motor Dysfunction Caused by PNI

The movement of muscles mainly depends on the innervation of nerves. After PNI, the muscle is denervated and begins to atrophy. As the denervated time increases, muscle mass, wet weight ratio, and muscle fiber cross-sectional area decrease and the structure becomes disordered, mainly manifested as amyosthenia and amyotrophy [11].

### 2.1. Behavioral Evidence

A large number of behavioral experiments have proved that tuina can improve the recovery of motor function of PNI rats. For example, the swash plate test proves that tuina can improve muscle strength and grasping ability [12, 13]. The TARLOV score proves that tuina can improve motor coordination [14]. The sciatic functional index (SFI), one of the indicators for evaluating fine motor, can also be significantly improved by tuina [15, 16].

### 2.2. Morphological Evidence

The wet weight ratio, fiber cross-sectional area, and diameter of muscle are the basic indicators of neuromuscular atrophy. After the tibial nerve is cut off, the wet weight ratio of the gastrocnemius muscle decreases significantly on the 7^th^ day and then slows down, and the cross-sectional area and diameter of muscle fiber decrease, while the wet weight ratio, cross-sectional area, and diameter can be increased after tuina intervention [17, 18]. Tuina can also increase the Muscle Atrophy Index (MAI), which is equal to muscle weight divided by body weight [15]. In order to explore the effects of tuina on spinal cord motor neurons, Li et al. [19] used a neuronal tract-tracing technique to observe the integrity of spinal cord motor neurons based on the sciatic nerve crush injury (SNI) model and observe CGRP-positive cells and microglia at the same time. It was found that the sciatic nerve injury stimulated CGRP-positive cells and activated the microglia in the ventral horn. After 20 times of intervention, the number of motor neurons in the tuina group was increased compared with that in the model group, similar to that in the normal group. The activity of CGRP-positive cells and microglia decreased, suggesting that tuina may promote the recovery of motor function by downregulating the activity of CGRP-positive cells and microglia in the ventral horn. By observing the ultrastructure of motor conduction pathway from the spinal cord to the peripheral, Yang [20] found that 28 days after modeling, in the SNI model group, the nucleoli of motor neurons in the ventral horn of the spinal cord were obviously pyknotic, the nuclear membrane was uneven, autophagosomes and lysosomes could be seen in the cytoplasm, the myelin sheath of the nerve injury point collapsed severely, axons atrophied seriously, gastrocnemius fibers arranged disorderly, and transverse striations disappeared. However, the number of autophagosomes and lysosomes in the tuina group was increased, the myelin sheath was relatively complete, the axons were not swollen or atrophied, gastrocnemius fibers arranged orderly and transverse striations were present, which showed that tuina could protect the stability of the ultrastructure of motor neurons, axons, and muscle fibers in the motor pathway and promote the production of autophagosomes.

### 2.3. Synaptic Plasticity

The synapse is the structure in which the axon terminals of neurons contact other neurons or nonneuronal cells. It is the place where neurons connect in function and a key part of information transmission occurs. Synaptic plasticity refers to the characteristic or phenomenon that the morphology and function of synapses can be changed over time. The effects of tuina on synaptic plasticity are mainly reflected in the following aspects. First of all, tuina can enhance the transcription of the synapsin I gene and the expression of p-synapsin I protein in the spinal cord of rats with SNI. Synapsin I has obvious specificity in synaptic formation and maturation and is an ideal indicator to measure synaptic plasticity [21]. Furthermore, Pan et al. [15] showed that tuina can inhibit the expression of tPA and PAI-1 in rats with a sciatic nerve crush injury (*P* < 0.05). Tissue plasminogen activator (tPA) is a kind of plasminogen activator, whose function is to catalyze the inactive plasminogen into active plasmin. Fibrin deposition aggravates myelin damage after sciatic nerve injury. tPA produced by Schwann cells (SCS) can increase the proteolytic activity of the fibrinolytic system, thereby removing fibrin deposited in the myelin sheath, promoting axon regeneration, and synaptic remodeling [22]. Tuina can shorten the pathological process of high expression of tPA and PAI-1, accelerate the dissolution of fibrin deposits, and create a good microenvironment for synaptic remodeling. Furthermore, the increased expression of inducible factors in the Agrin/MuSK signaling pathway by tuina may further promote the replacement of *γ*-*ε* subunit in neuromuscular junction (NMJ) development, promote neuromuscular reinnervation, and improve the transmission efficiency of synapses [21].

### 2.4. Autophagy

Autophagy is a cell's process of self-metabolism in inactive cytoplasm and organelles [23]. The first process of autophagy is to form an autophagosome precursor with a double-layer membrane structure, which gradually extends to engulf and wrap inactivated proteins and organelles, forming an autophagosome. Then, the autophagosome is transported to a lysosome through the cytoskeleton microtubule system, to form an autolysosome, which is then acidified to complete the metabolic process [24, 25]. Autophagy can remove excessive or damaged organelles and proteins in cells, participate in antigen presentation, and resist microbial intracellular infections, which play an important role in maintaining protein metabolic balance and intracellular environment homeostasis and is related to neurodegenerative diseases, cardiovascular and cerebrovascular diseases, tumors, and so forth [26]. Sensory and motor neurons, glial cells, and SCS in the nervous system all remove aging cell debris and inactivated proteins through autophagy [27]. Autophagy is also indispensable in the immune and inflammatory reactions within the spinal cord and at nerve injury points after PNI [28]. To explore the regeneration of PNI and the recovery of motor function by autophagy, Huang et al. [29] used the autophagy inducer rapamycin and autophagy inhibitor 3-methyladenine to treat rats with sciatic nerve injury. The results showed that rapamycin increased the number of autophagosomes and the expression of LC3-II, which was contrary to the treatment of autophagy inhibitors. It was demonstrated that autophagy can promote the regeneration of nerve axons, the repair of myelin, and the recovery of motor function. Another study showed that tuina could promote autophagy of spinal ventral horn neurons through the AMPK-mTOR pathway, accelerate the removal and reuse of necrotic organelles from nerve injury points and denervated muscle cells, and slow down muscle atrophy [20]. Tuina can also upregulate the mRNA expression of autophagy-related factors beclin-1, vacuolar protein sorting (vps34), and microtubule-associated protein light chain 3 (LC3) in the gastrocnemius muscle and enhance autophagy [20].

In addition to regulating synaptic plasticity and autophagy, tuina can also improve motor function in other ways. For example, Kong and Yan [30] found that tuina can relieve skeletal muscle atrophy by upregulating the positive expression area of the basic fibroblast growth factor (bFGF). Fu et al. [31] found that tuina can upregulate the expression of Desmin in the gastrocnemius of rats after sciatic nerve transection.

## 3. Tuina Can Improve Sensory Dysfunction as well as Improving Neuropathic Pain Caused by PNI

The body senses external stimuli through the receptors and then transmits that information to the sensory area of the cerebral cortex through the afferent nerve to generate sensation. PNI can cause pain, temperature, touch, and other sensory dysfunction, mainly manifested as pain, numbness, skin temperature sense, and tactile decline. Sensory dysfunction is the most common chief complaint in patients with PNI. Tuina plays an obvious role in relieving pain, promoting the recovery of temperature sensation and tactile disturbance, and so forth.

Neuropathic pain (NPP) is one of the main symptoms caused by PNI, mainly manifested as hyperalgesia, spontaneous pain, abnormal sensation, motor dysfunction, autonomic dysfunction, and so forth[32]. Long-term pain not only affects the sleep, life, and work of patients but also increases the incidence of depression, anxiety, and other emotional disorders, which seriously affects the quality of life of millions of people worldwide [33].

### 3.1. Behavioral Evidence

Cold hypersensitivity, one of the main clinical symptoms of patients with PNI, refers to the increased sensitivity and pain to cold stimulation which usually does not cause pain. Wu et al. [34] used ZH-6C intelligent cold and hot plate pain meter to observe the times of foot lifting occurring on the cold plate (3°C∼5°C) to evaluate the cold hypersensitivity. They found that the number of foot lifting events in 14 days and 21 days after tuina was less than that in the model group (*P* < 0.05), indicating that tuina can help the cold hyperesthesia caused by PNI return to normal.

PWL, PWT, cumulative pain scores, and hyperalgesia scores are the main behavioral indicators used to evaluate the degree of pain in animal experiments. The chronic constrictive injury (CCI) model can reduce PWL and PWT of NPP rats, while tuina can increase them and has a cumulative effect over time [35], which is consistent with the results in the spinal nerve ligation (SNL) model [36, 37]. On the 7^th^ day after modeling, the PWL and cumulative pain score of rats with SNI were significantly increased. After 20 days of intervention, the PWL and cumulative pain score were significantly decreased [38, 39]. Xian et al. [40] found that tuina intervention on SNI rats through a tuina manipulation simulator can significantly reduce the PWL and hyperalgesia scores. The CatWalk three-dimensional gait analyzer evaluates gait changes induced by pain through 5 parameters: print area (cm^2^), stand time (s), swing time (s), swing speed (cm/s), and max contact area (mm^2^). It has been shown that tuina can significantly improve the above parameters, suggesting that tuina has a better effect on improving the gait of rats with NPP [41].

### 3.2. Morphological Evidence

Microglia plays an important role in the generation and maintenance of pain [42]. After nerve injury, nociceptors are activated, and signals are transmitted to the spinal cord through A*δ* fibers and C fibers, activating microglia and multiple cascade reactions in microglia, and releasing IL-1*β*, IL-6, TNF-*α*, and many other inflammatory cytokines, which cause pain [43]. It has been preliminarily shown that tuina may exert analgesic effects by inhibiting the activation of microglia. Mo et al. [39] intervened SNI rats using three methods and three acupoints. They observed the activation degree of microglia in the spinal dorsal horn by immunofluorescence staining and found that the microglia in the model group were partially or fully activated. However, the microglia in the tuina group were partially activated or unactivated, which suggests that tuina can inhibit the activation of microglia. Ma [44] observed the morphological changes of the sciatic nerve by HE stain and found that seven days after modeling, the sciatic nerve in the model group was seen to be irregular in shape and fiber arrangement under a 400-fold light microscope, severe axonal edema and disordered arrangement can be seen in the model group after 20 days of intervention, while the arrangement of axons and fibers was neat and orderly in the tuina group.

### 3.3. Molecular Mechanism

Peripheral sensitization and central sensitization are the main mechanisms of pain [45, 46]. After PNI, the injured cells, mast cells, lymphocytes, and other inflammatory cells will release 5-hydroxytryptamine (5-HT), prostaglandin (PG), bradykinin, cytokines, and other inflammatory mediators, resulting in increased nociceptor sensitivity. Tuina can reduce the expression of inflammatory mediators and inhibit the sensitization process to achieve analgesic effects. For example, tuina can inhibit the expression of TNF-*α*, IL-1, and IL-1*β* in the serum of patients with lumbar disc herniation [47], downregulate 5-HT and PG in the serum of patients with sciatica [48], decrease the expression of proinflammatory factor IL-6 in serum and spinal cord of CCI model, and increase the expression of anti-inflammatory protective factor SOCS3 [38].

Wei et al. [49] demonstrated that tuina can downregulate the expression of Raf-1-ERK-CREB genes and proteins in the ERK signaling pathway, as well as downregulating phosphorylated pMAPK in the spinal cord, thereby inhibiting the expression of downstream inflammatory factor IL-1*β* and blocking the inflammatory response. Tuina also inhibits the release of inflammatory factors by regulating the TLR4 signaling pathway [36].

Pain is closely related to the imbalance of excitatory neurotransmitters and inhibitory neurotransmitters. Elevated levels of excitatory neurotransmitters will cause primary neuron depolarization, leading to increased sensitivity of nociceptive sensory neurons, while decreased levels of inhibitory neurotransmitters will lead to the generation or exacerbation of pain. Substance P (SP) is a neuropeptide widely distributed in central and peripheral tissues and has pain-causing effects. When the C-afferent fibers are selectively excited by noxious stimulation, SP release is induced and NK-1 receptor is activated, causing depolarization of dorsal horn neurons, thus transmitting pain. It has been shown that tuina can downregulate the content of SP in DRG but has no obvious effect in serum and spinal dorsal horn [41, 50]. To explore whether tuina analgesia was related to the inhibitory neurotransmitters 5-HT_2A_ receptor and GABA-B receptor in the spinal cord, Tao [51] intervened CCI model with dial method for 20 times and found that there was no significant difference in the level of these two receptors between the dial method group and the normal group, which suggests that tuina can upregulate the expression of 5-HT_2A_ receptor and GABA-B receptor, reduce the central disinhibition effect, reduce the transmission of nociceptive information, and ultimately alleviate spontaneous pain of NPP. CGRP is an important neurotransmitter related to pain formation and maintenance, which is widely distributed in nerve tissue. CGRP is a very important substance in nerve regeneration whose expression changes after PNI may participate in the early transmission of injury signals to the central nervous system and activate various pathways to induce nerve regeneration [52]. Tuina can significantly promote the release of CGRP. It is speculated that the mechanism by which tuina promotes nerve injury recovery is to increase the release of CGRP in DRG, promote the regeneration and survival of sensory neurons, and promote the recovery of sensory function [40]. In addition, tuina can also achieve analgesic effects by regulating the expression of P2X3 [53, 54], *β*-EP [55, 56], and other neurotransmitters.

In recent years, many studies have found that the production of NPP is related to autophagy in the spinal dorsal horn [57]. Deng [37] found that the expression of *sequestosomel* (p62) and LC3 increased in the spinal dorsal horn when autophagy was induced, which shows that autophagy in the dorsal horn of the spinal cord is related to NPP. At the same time, she also found that tuina could significantly increase the levels of p62 and LC3, suggesting that the analgesic mechanism of tuina may be related to the upregulation of spinal autophagy and inhibition of the inflammatory reaction.

## 4. Tuina Can Improve the Proliferation of Schwann Cell as well as Promoting the Regeneration of Myelin Sheath and Axon

Schwann cells (SCS), glial cells that form myelin in the PNS, are the main structural and functional cells of peripheral nerves, which can synthesize and secrete a variety of neurotrophic factors and adhesion molecules. The proliferation of SCS is essential for axonal regeneration, myelin formation, and nerve regeneration [58]. Peripheral nerve grafts with SCS removed have limited ability to repair nerve defects, especially long-segment nerve defects.

Peripheral myelin basic protein (MBP) is a strong basic protein synthesized and secreted by SCS, which plays a priming role in the process of myelin formation. Laminin (LN) can maintain the stability of the growth cone, promote the synthesis of extracellular matrix components by SCS, and play a role in its adhesion process. Cui [59] used immunohistochemistry combined with HE staining to prove that tuina can increase the expression of LN in spinal cord and nerve injury points, promote the proliferation of SCS, decrease the expression of MBP, reduce the shedding of the myelin sheath, and promote the repair of myelin.

Shen et al. [13] used transmission electron microscopy to observe that 20 days after modeling, part of the myelin sheath was severely collapsed in the model group, while part of the myelin sheath phospholipid fell off and myelin sheath collapse occurred only occasionally in the tuina group, which suggests that tuina can reduce the deformation of the myelin sheath and promote the repair and regeneration of myelinated fibers. G-ratio is the ratio of the axon diameter to the total diameter of myelinated fibers, reflecting the thickness of the myelin sheath. The larger the value, the thinner the myelin sheath. On the 20^th^ day after the intervention, the g-ratio in the tuina group was higher than that in the model group (*P* < 0.05), with no significant difference compared with the sham operation group (*P* > 0.05), indicating that tuina can reduce myelin thickness. At the same time, the expression of NRG1 and ErbB2 increased, suggesting that tuina could protect the survival of SCS and improve the deformation of myelin sheath by upregulating the expression of NRG1 and erbB2 in L4-6 spinal cords, thereby promoting nerve repair.

Transforming growth factor *β*1 (TGF-*β*1), as an important cytokine of SCS, can promote the transformation of SCS to an unmyelinated phenotype in the early stage of injury and promote SCS proliferation and scar formation. Shao et al. [60] quantitatively analyzed the SCS marker S100 at the injury point of the sciatic nerve and found that tuina can promote SCS proliferation and myelin repair. However, they did not find that tuina can affect the levels of TGF-*β*1 or Smad2. Therefore, it is speculated that the myelin repair effect of tuina may not be related to the TGF-*β*1/Smad2 pathway. Lu [61] found that tuina can significantly promote the regeneration of myelin in SNI rats and promote the secretion of axon guiding factors Netrin 1 and Slit 2 by SCS.

The cyclic adenylic acid- (cAMP-) protein kinase (PKA) signaling pathway is involved in the repair process of PNI. Previous research has shown that the cAMP-PKA signaling pathway plays an important role in promoting axon and myelin regeneration, promoting NGF expression, and regulating growth cone guidance [62, 63]. Studies have shown that the levels of cAMP and PKA protein in DRG of rats with SNI decreased significantly, but after 20 times of intervention, the levels of both increased significantly, suggesting that tuina can activate the cAMP-PKA signaling pathway and help repair damaged nerves [64].

Neurotrophic factors (NTFs) are polypeptide molecules that can nourish nerve cells. SCS are an important source of NTF. Many experiments have demonstrated that tuina can promote the repair of injured nerves by promoting the expression of a variety of NTFs. For example, Mei et al. [65, 66] proved that tuina can promote the expression of NGF in the spinal cord of rats with sciatic nerve clamping injury, reduce the release of low-affinity receptor p75NTR, increase the release of high-affinity receptor TrkA, inhibit cell apoptosis, protect neurons, and promote nerve regeneration. Wang et al. [67] found that tuina can also promote the expression of NGF in rats after sciatic nerve anastomosis, but the effect on SCS was not obvious. The reason may be that, as the nerve is repaired, SCS will migrate to the place far away from the injury site, whereas the sampling site was close to the injury site. Yuan [68] demonstrated that tuina can upregulate the expression of nerve growth factor (NFG), brain-derived neurotrophic factor (BDNF), ciliary neurotrophic factor (CNTF), and basic fibroblast growth factor (bFCF). Geng et al. [69] proved that tuina can promote the expression of NT-3 and its receptor TrkC in DRG and ventral horn in SNI rats and promote the growth of neuron cell bodies and processes, thereby promoting nerve repair.

## 5. Tuina Can Promote Axoplasmic Transportation

Axoplasmic transportation is one of the characteristics of nerve cells. Nutrients in cells can be transported from the cell body to the distal end of axons, or vice versa. After PNI, if the axoplasmic transport function cannot be restored to normal in time, the effector will be denervated and atrophy, which will affect the motor function, the neuron will lose nourishment from the distal, and the function will be impaired. The recovery of axoplasmic transport function is an important index for evaluating nerve recovery. The power of axoplasmic transport comes from motor proteins, which include kinesin and dynein [70]. Tuina can upregulate the expression of kinesin and dynein, promote the recovery of axoplasmic transport functions, affect neurons and the target tissue in both directions, and ultimately improve the motor and sensory function of rats with sciatic nerve injury [71].

## 6. Conclusion

Current evidence shows that tuina can promote PNI recovery through a variety of ways, including autophagy, synaptic plasticity, axon regeneration, and remyelination, and ultimately achieve the purpose of restoring sensory and motor function.

Although the mechanism of tuina to promote the repair of PNI has made significant progress, there are still many limitations. For example, existing studies on the mechanism of tuina promoting PNI recovery are mostly limited to peripheral mechanisms, and there are few studies on central mechanisms. Xing et al. [72] used functional magnetic resonance imaging (fMRI) to explore the brain mechanism of tuina on the repair of PNI. Based on the sciatic nerve transection model, resting-state fMRI showed that the ALFF values in the left somatosensory cortex in the tuina group were higher than the model or sham-tuina group, suggesting that tuina can promote the adaptive changes in the somatosensory cortex and improve the recovery of local brain activity after peripheral nerve injury.

Moreover, current research technologies are relatively limited. More advanced technology should be introduced to explore the massage mechanism. To explore the influence of tuina on the gene level of PNI, Lv et al. [16] used RNA-Seq technology to detect the genetic changes of SNI rats between the model group and the tuina group. At the point of nerve injury, there were 221 differentially expressed genes (DEGs) between the two groups, which were mainly enriched in the biological processes related to the regulation of myocytes, such as the regulation of striated muscle cells differentiation, myoblast differentiation, and myotube differentiation. The genes most related to biological function are Myog and Myod1. In DRGs, there were 226 upregulated or downregulated DEGs, enriching in positive regulation, protein binding, response to pressure, and so forth. The metabolic pathways with significant differences included Wnt, IL-17, and MAPK signaling pathways [73].

There remains a large amount of unknown and uncertain fields that need to be further explored. More in vivo studies and clinical trials are needed to assess the therapeutic benefit of tuina in the treatment of PNI.

## References

[1] Palombella S., Guiotto M., Higgins G. C. (2020). Human platelet lysate as a potential clinical-translatable supplement to support the neurotrophic properties of human adipose-derived stem cells. *Stem Cell Research & Therapy*.

[2] Rigoni M., Negro S. (2020). Signals orchestrating peripheral nerve repair. *Cells*.

[3] Gordon T. (2016). Nerve regeneration in the peripheral and central nervous systems. *The Journal of Physiology*.

[4] Soman S. S., Vijayavenkataraman S. (2020). Perspectives on 3D bioprinting of peripheral nerve conduits. *International Journal of Molecular Sciences*.

[5] Neumann B., Linton C., Giordano-Santini R., Hilliard M. A. (2019). Axonal fusion: an alternative and efficient mechanism of nerve repair. *Progress in Neurobiology*.

[6] Xu Z., Orkwis J. A., DeVine B. M., Harris G. M. (2020). Extracellular matrix cues modulate Schwann cell morphology, proliferation, and protein expression. *Journal of Tissue Engineering and Regenerative Medicine*.

[7] Ma J. Q., Li F. G., Lv Z. G. (2018). Clinical observation of massage in the treatment of obstetric brachial plexus injury. *Chinese Journal of Traditional Medical Traumatology & Orthopedics*.

[8] Pach D., Piper M., Lotz F. (2018). Effectiveness and cost-Effectiveness of tuina for chronic neck pain: a randomized controlled trial comparing tuina with a no-Intervention waiting list. *The Journal of Alternative and Complementary Medicine*.

[9] Mo Z. M., Li D., Zhang R. W., Chang M., Yang B., Tang S. (2019). Comparisons of the effectiveness and safety of Tuina, acupuncture, traction, and Chinese herbs for lumbar disc herniation: a systematic review and network meta-analysis. *Evidence-Based Complementary and Alternative Medicine*.

[10] Kumar S., Beaton K., Hughes T. (2013). The effectiveness of massage therapy for the treatment of nonspecific low back pain: a systematic review of systematic reviews. *International Journal of General Medicine*.

[11] Gigo-Benato D., Russo T. L., Geuna S., Domingues N. R. S. R., Salvini T. F., Parizotto N. A. (2010). Electrical stimulation impairs early functional recovery and accentuates skeletal muscle atrophy after sciatic nerve crush injury in rats. *Muscle & Nerve*.

[12] Li Y. Z., Yang C., Yu T. Y. (2018). Effects of tuina of three handing-three points on motor function and expression of NogoA and its receptor in spinal cord of rats with sciatic nerve injury. *Chinese Journal of Rehabilitation Theory and Practice*.

[13] Shen Y., Mo Y. J., Yu T. Y. (2020). Effects of three handing-three points on thickness of myelin sheath and expression of neuregulin 1 and human epidermal growth factor receptor 2 after sciatic nerve injury in rats. *Chinese Journal of Rehabilitation Theory and Practice*.

[14] Wu J. C., Lu M. Q., Yu Y. (2014). Investigation of tuina therapy on NT-3 and TrkC of sciatic nerve injury model rats. *Chinese Archives of Traditional Chinese Medicine*.

[15] Pan F., Yu T.-y., Wong S. (2017). Chinese tuina downregulates the elevated levels of tissue plasminogen activator in sciatic nerve injured sprague-dawley rats. *Chinese Journal of Integrative Medicine*.

[16] Lv T. T., Mo Y. J., Yu T. Y. (2020). An investigation into the rehabilitative mechanism of tuina in the treatment of sciatic nerve injury. *Evidence-Based Complementary and Alternative Medicine*.

[17] An H. Y., Tang C. L., Huang S. Q. (2019). Effect of tuina on autophagy-related factor Beclin-1, vacuolar protein sorting and microtubule- associated protein light chain 3 in rats with denervated skeletal muscle atrophy. *Chinese Journal of Rehabilitation Theory and Practice*.

[18] Wan X. F., Tang C. L., Zhao D. D. (2019). Therapeutic effect of massage on denervated skeletal muscle atrophy in rats and its mechanism. *Chinese Journal of Applied Physiology*.

[19] Li Y. Z., Miao R. P., Yu T. Y. (2019). Mild mechanic stimulate on acupoints regulation of CGRP-positive cells and microglia morphology in spinal cord of sciatic nerve injured rats. *Frontiers in Integrative Neuroscience*.

[20] Yang C. (2018). Mechanism of three methods and three acupoints regulating autophagy to promote recovery of motor function in SNI rats.

[21] Pan F. (2015). Exploring the mechanism of massage improving the motor function of SNI model rats from the perspective of synaptic plasticity.

[22] Akassoglou K., Kombrinck K. W., Degen J. L., Strickland S. (2000). Tissue plasminogen activator-mediated fibrinolysis protects against axonal degeneration and demyelination after sciatic nerve injury. *Journal of Cell Biology*.

[23] Bento C. F., Renna M., Ghislat G. (2016). Mammalian autophagy: how does it work?. *Annual Review of Biochemistry*.

[24] Cadwell K. (2016). Crosstalk between autophagy and inflammatory signaling pathways: balancing defence and homeostasis. *Nature Reviews Immunology*.

[25] Sarkar S. (2013). Regulation of autophagy by mTOR-dependent and mTOR-independent pathways: autophagy dysfunction in neurodegenerative diseases and therapeutic application of autophagy enhancers. *Biochemical Society Transactions*.

[26] Mizushima N., Komatsu M. (2011). Autophagy: renovation of cells and tissues. *Cell*.

[27] Levine B., Klionsky D. J. (2004). Development by self-digestion: molecular mechanisms and biological functions of autophagy. *Developmental Cell*.

[28] Smith E. D., Prieto G. A., Tong L. (2014). Rapamycin and interleukin-1*β* impair brain-derived neurotrophic factor-dependent neuron survival by modulating autophagy. *Journal of Biological Chemistry*.

[29] Huang H.-c., Chen L., Zhang H.-x. (2016). Autophagy promotes peripheral nerve regeneration and motor recovery following sciatic nerve crush injury in rats. *Journal of Molecular Neuroscience*.

[30] Kong Y. M., Yan J. T. (2019). Study on the expression of basic fibroblast growth factor after skeletal muscle denervation by massage combined with running wheel training. *Chinese Manipulation and Rehabilitation Medicine*.

[31] Fu X., Zhang J., Song Z. (2018). Effect of tuina manipulation on the expression of desmin in gastrocnemius of rats with sciatic nerve injury. *Rehabilitation Medicine*.

[32] Liu Z. Y., Song Z. W., Guo S. W. (2019). CXCL12/CXCR4 signaling contributes to neuropathic pain via central sensitization mechanisms in a rat spinal nerve ligation model. *CNS Neuroscience & Therapeutics*.

[33] Tölle T. R. (2010). Challenges with current treatment of neuropathic pain. *European Journal of Pain Supplements*.

[34] Wu S., Yu T. Y., Yang C. (2017). A behavioral study of cold sensation hypersensitivity in sciatic nerve injury model rats using three methods and three points. *Guiding Journal of Traditional Chinese Medicine and Pharmacy*.

[35] Wei B. L. (2018). Study on the effect and mechanism of massage on rats with chronic neuropathic pain based on ERK signaling pathway.

[36] Liang Y. Y. (2019). *The Study on the Analgesic Mechanism of Pivot Meridian Massage on Neuropathic Pain Rats Based on TLR4 Signal Pathway Regulated by MiRNA-146a*.

[37] Deng M. Q. (2019). *The Effect of Spinal Dorsal Horn Autophagy on Neuropathic Pain in Rats and the Analgesic Mechanism of Massage*.

[38] Ma C., Yao B. B., Yu T. Y. (2017). Effects of bofa on expresion of IL-6 and SOCS3 in spinal cord in CCI rats. *Journal of Nanjing University of Traditional Chinese Medicine*.

[39] Mo Y. J., Zhang Y. M., Yu T. Y. (2020). Effect of three handing-three points on pain function and expression of CX3CL1/CX3CR1 in spinal cord dorsal horn of rats with sciatic nerve injury. *Chinese Journal of Rehabilitation Theory and Practice*.

[40] Xian S. T., Xian T. Y., Yu T. Y. (2015). Effects of massage on the expression of calcitonin gene-related peptide in dorsal root ganglion of chronic constriction injury rats. *Acta Chinese Medicine*.

[41] Zhao D. (2019). *Effects of Analgesic and SP/NK-1R Signaling Pathway in Dorsal Root Ganglia on CCI Model Rats by Tuina*.

[42] Aldskogius H., Kozlova E. (2013). Microglia and neuropathic pain. *CNS & Neurological Disorders—Drug Targets*.

[43] Arroyo D. S., Gaviglio E. A., Peralta Ramos J. M., Bussi C., Rodriguez-Galan M. C., Iribarren P. (2014). Autophagy in inflammation, infection, neurodegeneration and cancer. *International Immunopharmacology*.

[44] Ma C. (2017). Study on the mechanism of anti-inflammatory and analgesic effect of bo manipulation on CCI rats.

[45] Xing F., Gu H. W., Niu Q. (2019). MZF1 in the dorsal root ganglia contributes to the development and maintenance of neuropathic pain via regulation of TRPV1. *Neural Plasticity*.

[46] Cohen S. P., Mao J. (2014). Neuropathic pain: mechanisms and their clinical implications. *BMJ*.

[47] Yang J. K., Wang J. W., Zhou K. W. (2020). Study on the curative effect of Duhuzhongtang combined with massage in patients with lumbar disc herniation and the changes of TXB2, TNF-*α*, IL-1*β*,. *Chinese Archives of Traditional Chinese Medicine*.

[48] Chen M. R., Huang Y. H. (2013). Effect on 5-HT, prostaglandin and calcium ion in sciatica by drafting fascia to relieve spasm of massage: a clinical observation of 50 cases. *Guiding Journal of Traditional Chinese Medicine and Pharmacy*.

[49] Wei B. L., Tang H. L., Wang X. J. (2018). The effect of massage on the expression of phosphorylated P38MAPK and the inflammatory factor IL-1*β* in the spinal cord of rats with neuropathic pain. *Lishizhen Medicine and Materia Medica Research*.

[50] Lin Z. G., Chen S. J., Chen L. C. (2019). Analgesic effect of tuina pressing and kneading method on neuropathic pain rats and its effect on SP in serum, dorsal root ganglia and spinal dorsal horn. *Chinese Archives of Traditional Chinese Medicine*.

[51] Tao Y. H. (2017). *Effects on Bofa on the Expression of Inhibitory Neurotransmitters and Inflammatory Factors in CCI Rats*.

[52] Seybold V. S., Mccarson K. E., Mermelstein P. G., Groth R. D., Abrahams L. G. (2003). Calcitonin gene-related peptide regulates expression of Neurokinin1Receptors by rat spinal neurons. *The Journal of Neuroscience*.

[53] Lin Z. G., Jiang S. C., Cheng Y. B. (2017). Experimental study of tuina on DRG neurons P2X3 receptor of lumbar disc herniation rats. *Chinese Archives of Traditional Chinese Medicine*.

[54] Chen L. C., Lin Z. G. (2019). The effect of massage on the expression of P2X3 receptor in dorsal root ganglion of rats with neuropathic pain. *Lishizhen Medicine and Materia Medica Research*.

[55] Chen Y. W., Li P., Zhang Z. B. (2016). Experimental study on the effect and mechanism of manipulation on CCI model in rats. *Rheumatism and Arthritis*.

[56] Li Z. Y., Yu B. Y., Jin Y. (2008). Pressing and kneading on Huantiao(GB30): analgesic effect and central mechanism of rats with neuropathic pain. *Liaoning Journal of Traditional Chinese Medicine*.

[57] Berliocchi L., Russo R., Maiaru M. (2011). Autophagy impairment in a mouse model of neuropathic pain. *Molecular Pain*.

[58] Huang Z., Powell R., Phillips J. B., Haastert-Talini K. (2020). Perspective on schwann cells derived from induced pluripotent stem cells in peripheral nerve tissue engineering. *Cells*.

[59] Cui M. Z. (2015). Effect of massage on the expression of MBP and LN in spinal cord and nerve of SNI rats.

[60] Shao S., Mo Y. J., Yu T. Y. (2020). Effects of three handing-three points on motor function and expression of transforming growth factor-*β*1/Smad2 pathway in injured sciatic nerve of rats. *Chinese Journal of Rehabilitation Theory and Practice*.

[61] Lu M. Q. (2016). Effect of tuina on the guiding mechanism of axon growth during nerve repair and regeneration in SNI rats.

[62] Cui Q. i., Yip H. K., Zhao R. C. H., So K.-F., Harvey A. R. (2003). Intraocular elevation of cyclic AMP potentiates ciliary neurotrophic factor-induced regeneration of adult rat retinal ganglion cell axons. *Molecular and Cellular Neuroscience*.

[63] Chierzi S., Ratto G. M., Verma P., Fawcett J. W. (2005). The ability of axons to regenerate their growth cones depends on axonal type and age, and is regulated by calcium, cAMP and ERK. *European Journal of Neuroscience*.

[64] Luo Y. T., Mo Y. J., Lv T. T. (2020). Effects of “three methods and acupoints” on the expression of cAMP and PKA in dorsal root ganglion of rats with sciatic nerve injury. *Global Traditional Chinese Medicine*.

[65] Mei X. H., Ji Q., Yao Binbin B. B. (2013). Investigation of tuina therapy on NGF and p75NTR of sciatic nerve injury model rats. *China Journal of Traditional Chinese Medicine and Pharmacy*.

[66] Mei X. H., Ji Q., Wu J. C. (2013). Influences of tuina therapy on nerve growth factor and TrkA receptor of NGF in rats with sciatic nerve injury. *Journal of Beijing University of Traditional Chinese Medicine*.

[67] Wang C. H., Yan J. T., Ma S. J. (2018). Effects of tuina combined with treadmill training on schwann cells and related neurotrophic factors after sciatic nerve anastomosisin rats. *Journal of New Chinese Medicine*.

[68] Yuan Z. L. (2011). Effect of Tuina on NFTs in rats with sciatic nerve injury.

[69] Geng N., Xian S. T., Wu J. C. (2014). Tuina’s effect on NT-3/TrkC of sciatic nerve injury rats. *Journal of Nanjing University of Traditional Chinese Medicine*.

[70] Dixit R., Ross J. L., Goldman Y. E., Holzbaur E. L. F. (2008). Differential regulation of dynein and kinesin motor proteins by tau. *Science*.

[71] Yao B. B., Mei X. H., Wu J. C. (2013). Stud_y_ on the influence of massage to sciatic nerve injured rat’s axoplasmic transport function based on motor protein. *Journal of Nanjing University of Traditional Chinese Medicine*.

[72] Xing X. X., Zheng M. X., Hua X. Y. (2021). Brain plasticity after peripheral nerve injury treatment with tuina therapy based on resting-state functional magnetic resonance imaging. *Neural Regeneration Research*.

[73] Lv T. T., Mo Y. J., Yu T. Y. (2020). Using RNA-Seq to explore the repair mechanism of the three methods and three-acupoint technique on DRGs in sciatic nerve injured rats. *Pain Research and Management*.

